# Supplementary material for: Structure of the human volume regulated anion channel
Source: eLife. 2018 Aug 10;7:e38461. doi: 10.7554/eLife.38461 (PMC6086657; doi:10.7554/eLife.38461)
Supplement: Reporting standard 1 [file elife-38461-fig3.pdf]

Full wwPDB/EMDataBank EM Map/Model Validation Report (i)

Jul 12, 2018 – 09:35 AM EDT

PDB ID	:	6DJB
EMDB ID:	:	EMD-7935
Title	:	Structure of human Volume Regulated Anion Channel composed of SWELL1 (LRRC8A)
Deposited on	:	2018-05-24
Resolution	:	4.40 Å(reported)

This is a Full wwPDB/EMDataBank EM Map/Model Validation Report.

This report is produced by the wwPDB biocuration pipeline after annotation of the structure.

We welcome your comments at *validation@mail.wwpdb.org*
A user guide is available at
https://www.wwpdb.org/validation/2017/EMValidationReportHelp
with specific help available everywhere you see the (i) symbol.

MolProbity	:	4.02b-467
Percentile statistics	:	20171227.v01 (using entries in the PDB archive December 27th 2017)
Ideal geometry (proteins)	:	Engh & Huber (2001)
Ideal geometry (DNA, RNA)	:	Parkinson et. al. (1996)
Validation Pipeline (wwPDB-VP)	:	rb-20031172

CONFIDENTIAL VALIDATION REPORT

1 Overall quality at a glance (i)

The following experimental techniques were used to determine the structure:
ELECTRON MICROSCOPY

The reported resolution of this entry is 4.40 Å.

Percentile scores (ranging between 0-100) for global validation metrics of the entry are shown in the following graphic. The table shows the number of entries on which the scores are based.

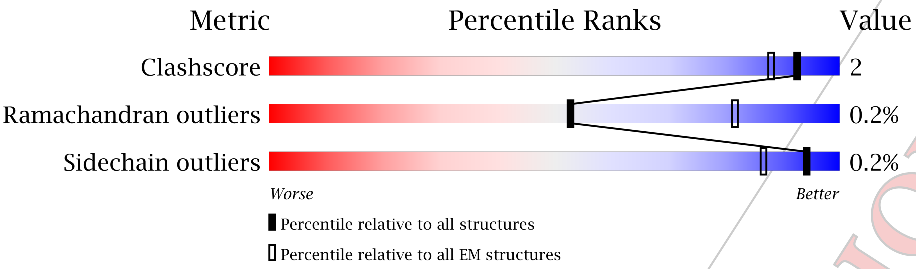

Metric	Whole archive (#Entries)	EM structures (#Entries)
Clashscore	136327	1886
Ramachandran outliers	132723	1663
Sidechain outliers	132532	1531

The table below summarises the geometric issues observed across the polymeric chains. The red, orange, yellow and green segments on the bar indicate the fraction of residues that contain outliers for >=3, 2, 1 and 0 types of geometric quality criteria. A grey segment represents the fraction of residues that are not modelled. The numeric value for each fraction is indicated below the corresponding segment, with a dot representing fractions <=5%

Mol	Chain	Length	Quality of chain
1	A	810	82% 5% 13%
1	B	810	87% • 10%
1	C	810	82% 5% 13%
1	D	810	87% • 10%
1	E	810	82% 5% 13%
1	F	810	87% • 10%

CONFIDENTIAL VALIDATION REPORT

2 Entry composition (i)

There is only 1 type of molecule in this entry. The entry contains 34707 atoms, of which 0 are hydrogens and 0 are deuteriums.

In the tables below, the AltConf column contains the number of residues with at least one atom in alternate conformation and the Trace column contains the number of residues modelled with at most 2 atoms.

- Molecule 1 is a protein called Volume-regulated anion channel subunit LRRC8A.

Mol	Chain	Residues	Atoms					AltConf	Trace
			Total	C	N	O	S		
1	A	706	5739	3735	972	1007	25	0	0
1	B	725	5830	3788	991	1026	25	0	0
1	E	706	5739	3735	972	1007	25	0	0
1	F	725	5830	3788	991	1026	25	0	0
1	C	706	5739	3735	972	1007	25	0	0
1	D	725	5830	3788	991	1026	25	0	0

CONFIDENTIAL VALIDATION REPORT

3 Residue-property plots ⓘ

These plots are drawn for all protein, RNA and DNA chains in the entry. The first graphic for a chain summarises the proportions of the various outlier classes displayed in the second graphic. The second graphic shows the sequence view annotated by issues in geometry. Residues are color-coded according to the number of geometric quality criteria for which they contain at least one outlier: green = 0, yellow = 1, orange = 2 and red = 3 or more. Stretches of 2 or more consecutive residues without any outlier are shown as a green connector. Residues present in the sample, but not in the model, are shown in grey.

- Molecule 1: Volume-regulated anion channel subunit LRRC8A

Chain A:

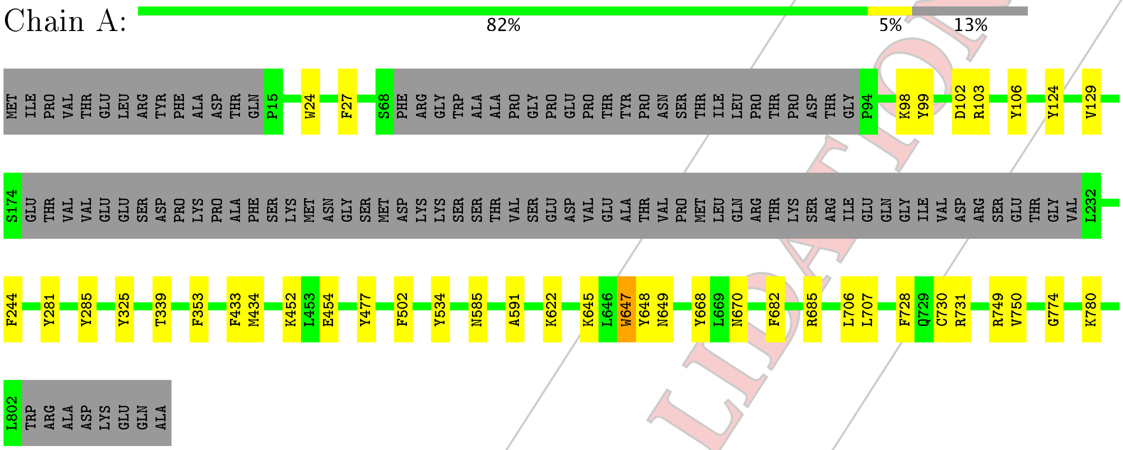

- Molecule 1: Volume-regulated anion channel subunit LRRC8A

Chain B:

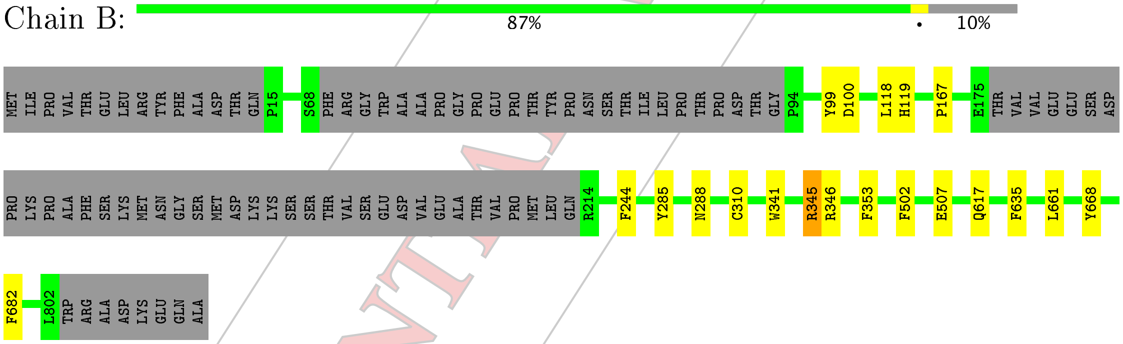

- Molecule 1: Volume-regulated anion channel subunit LRRC8A

Chain E:

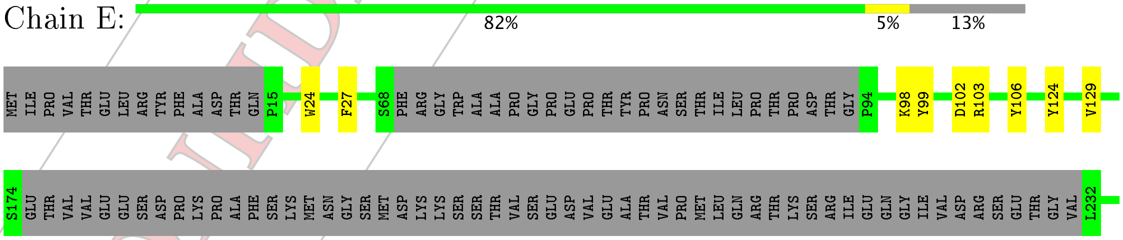

CONFIDENTIAL VALIDATION REPORT

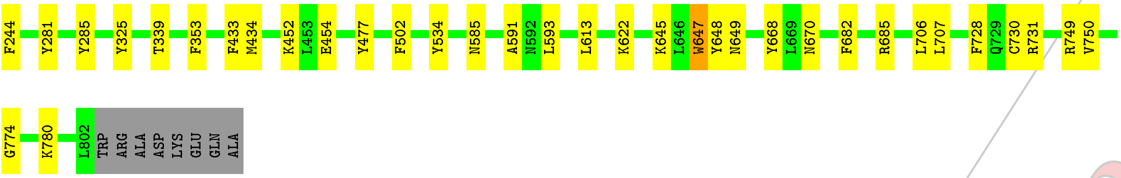

• Molecule 1: Volume-regulated anion channel subunit LRRC8A

Chain F:

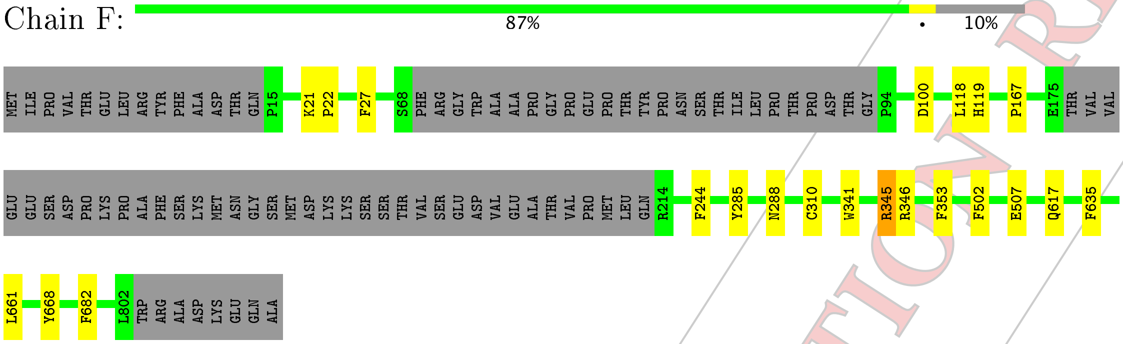

• Molecule 1: Volume-regulated anion channel subunit LRRC8A

Chain C:

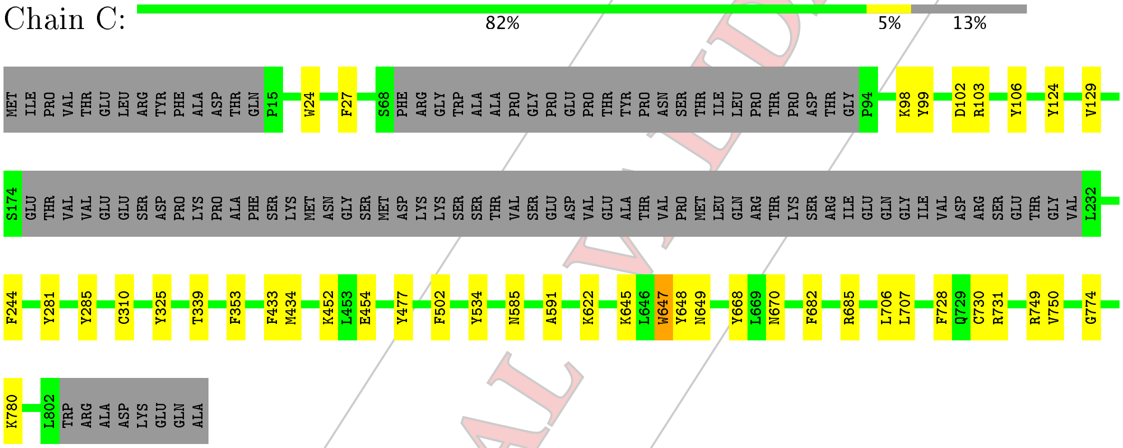

• Molecule 1: Volume-regulated anion channel subunit LRRC8A

Chain D:

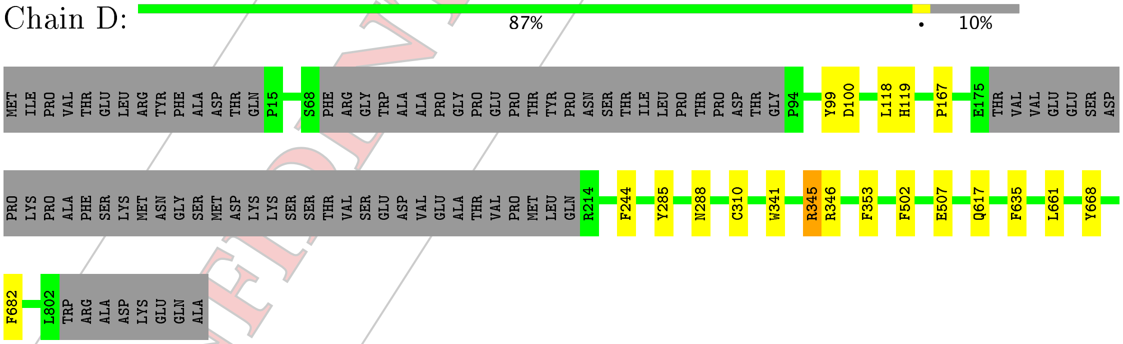

CONFIDENTIAL VALIDATION REPORT

4 Experimental information (i)

Property	Value	Source
Reconstruction method	SINGLE PARTICLE	Depositor
Imposed symmetry	POINT, C3	Depositor
Number of particles used	25719	Depositor
Resolution determination method	FSC 0.143 CUT-OFF	Depositor
CTF correction method	PHASE FLIPPING AND AMPLITUDE CORRECTION	Depositor
Microscope	FEI TALOS ARCTICA	Depositor
Voltage (kV)	200	Depositor
Electron dose $(e^-/\text{Å}^2)$	55	Depositor
Minimum defocus (nm)	800	Depositor
Maximum defocus (nm)	1500	Depositor
Magnification	36000	Depositor
Image detector	GATAN K2 SUMMIT (4k x 4k)	Depositor

CONFIDENTIAL VALIDATION REPORT

5 Model quality (i)

5.1 Standard geometry (i)

The Z score for a bond length (or angle) is the number of standard deviations the observed value is removed from the expected value. A bond length (or angle) with $|Z| > 5$ is considered an outlier worth inspection. RMSZ is the root-mean-square of all Z scores of the bond lengths (or angles).

Mol	Chain	Bond lengths		Bond angles	
		RMSZ	#|Z| >2	RMSZ	#|Z| >2
1	A	0.65	0/5865	0.89	13/7955 (0.2%)
1	B	0.64	1/5956 (0.0%)	0.86	5/8081 (0.1%)
1	C	0.65	1/5865 (0.0%)	0.89	13/7955 (0.2%)
1	D	0.64	1/5956 (0.0%)	0.86	5/8081 (0.1%)
1	E	0.65	0/5865	0.89	13/7955 (0.2%)
1	F	0.64	1/5956 (0.0%)	0.86	5/8081 (0.1%)
All	All	0.65	4/35463 (0.0%)	0.88	54/48108 (0.1%)

All (4) bond length outliers are listed below:

Mol	Chain	Res	Type	Atoms	Z	Observed(Å)	Ideal(Å)
1	F	310	CYS	CB-SG	-7.91	1.68	1.82
1	B	310	CYS	CB-SG	-7.89	1.68	1.82
1	D	310	CYS	CB-SG	-7.87	1.68	1.82
1	C	310	CYS	CB-SG	-5.02	1.73	1.81

All (54) bond angle outliers are listed below:

Mol	Chain	Res	Type	Atoms	Z	Observed(°)	Ideal(°)
1	E	502	PHE	CB-CG-CD2	-7.10	115.83	120.80
1	C	502	PHE	CB-CG-CD2	-7.09	115.84	120.80
1	A	502	PHE	CB-CG-CD2	-7.09	115.84	120.80
1	E	106	TYR	CB-CG-CD2	-6.77	116.94	121.00
1	A	106	TYR	CB-CG-CD2	-6.74	116.96	121.00
1	C	106	TYR	CB-CG-CD2	-6.71	116.98	121.00
1	A	285	TYR	CB-CG-CD2	-5.93	117.44	121.00
1	C	285	TYR	CB-CG-CD2	-5.92	117.45	121.00
1	C	353	PHE	CB-CG-CD1	5.91	124.93	120.80
1	E	285	TYR	CB-CG-CD2	-5.87	117.48	121.00
1	A	353	PHE	CB-CG-CD1	5.81	124.87	120.80
1	E	353	PHE	CB-CG-CD1	5.81	124.87	120.80
1	F	244	PHE	CB-CG-CD2	-5.78	116.75	120.80
1	B	244	PHE	CB-CG-CD2	-5.78	116.75	120.80

Continued on next page...

CONFIDENTIAL VALIDATION REPORT

Continued from previous page...

Mol	Chain	Res	Type	Atoms	Z	Observed(^{o})	Ideal(^{o})
1	D	244	PHE	CB-CG-CD2	-5.76	116.76	120.80
1	B	285	TYR	CB-CG-CD2	-5.76	117.55	121.00
1	F	285	TYR	CB-CG-CD2	-5.73	117.56	121.00
1	D	285	TYR	CB-CG-CD2	-5.70	117.58	121.00
1	C	353	PHE	CB-CG-CD2	-5.60	116.88	120.80
1	F	353	PHE	CB-CG-CD2	-5.57	116.90	120.80
1	A	353	PHE	CB-CG-CD2	-5.54	116.92	120.80
1	E	353	PHE	CB-CG-CD2	-5.51	116.94	120.80
1	B	353	PHE	CB-CG-CD2	-5.51	116.94	120.80
1	E	99	TYR	CB-CG-CD2	-5.51	117.70	121.00
1	A	99	TYR	CB-CG-CD2	-5.49	117.71	121.00
1	C	99	TYR	CB-CG-CD2	-5.46	117.72	121.00
1	A	668	TYR	CB-CG-CD2	-5.45	117.73	121.00
1	C	27	PHE	CB-CG-CD2	-5.45	116.99	120.80
1	D	353	PHE	CB-CG-CD2	-5.45	116.99	120.80
1	C	668	TYR	CB-CG-CD2	-5.44	117.73	121.00
1	A	27	PHE	CB-CG-CD2	-5.42	117.01	120.80
1	E	27	PHE	CB-CG-CD2	-5.41	117.01	120.80
1	E	668	TYR	CB-CG-CD2	-5.38	117.77	121.00
1	B	668	TYR	CB-CG-CD2	-5.30	117.82	121.00
1	F	668	TYR	CB-CG-CD2	-5.30	117.82	121.00
1	E	124	TYR	CB-CG-CD2	-5.30	117.82	121.00
1	D	668	TYR	CB-CG-CD2	-5.27	117.84	121.00
1	A	124	TYR	CB-CG-CD2	-5.26	117.84	121.00
1	E	281	TYR	CB-CG-CD2	-5.25	117.85	121.00
1	A	281	TYR	CB-CG-CD2	-5.25	117.85	121.00
1	C	124	TYR	CB-CG-CD2	-5.24	117.86	121.00
1	C	281	TYR	CB-CG-CD2	-5.23	117.86	121.00
1	C	244	PHE	CB-CG-CD2	-5.23	117.14	120.80
1	A	244	PHE	CB-CG-CD2	-5.22	117.15	120.80
1	E	244	PHE	CB-CG-CD2	-5.21	117.15	120.80
1	E	648	TYR	CB-CG-CD2	-5.15	117.91	121.00
1	E	534	TYR	CB-CG-CD2	-5.14	117.91	121.00
1	C	534	TYR	CB-CG-CD2	-5.09	117.94	121.00
1	A	648	TYR	CB-CG-CD2	-5.09	117.94	121.00
1	A	534	TYR	CB-CG-CD2	-5.06	117.97	121.00
1	D	99	TYR	CB-CG-CD2	-5.05	117.97	121.00
1	C	648	TYR	CB-CG-CD2	-5.04	117.98	121.00
1	B	99	TYR	CB-CG-CD2	-5.03	117.98	121.00
1	F	27	PHE	CB-CG-CD2	-5.02	117.29	120.80

There are no chirality outliers.

CONFIDENTIAL VALIDATION REPORT

There are no planarity outliers.

5.2 Too-close contacts (i)

In the following table, the Non-H and H(model) columns list the number of non-hydrogen atoms and hydrogen atoms in the chain respectively. The H(added) column lists the number of hydrogen atoms added and optimized by MolProbity. The Clashes column lists the number of clashes within the asymmetric unit, whereas Symm-Clashes lists symmetry related clashes.

Mol	Chain	Non-H	H(model)	H(added)	Clashes	Symm-Clashes
1	A	5739	0	5832	22	0
1	B	5830	0	5865	14	0
1	C	5739	0	5832	22	0
1	D	5830	0	5865	14	0
1	E	5739	0	5832	23	0
1	F	5830	0	5865	15	0
All	All	34707	0	35091	107	0

The all-atom clashscore is defined as the number of clashes found per 1000 atoms (including hydrogen atoms). The all-atom clashscore for this structure is 2.

All (107) close contacts within the same asymmetric unit are listed below, sorted by their clash magnitude.

Atom-1	Atom-2	Interatomic distance (Å)	Clash overlap (Å)
1:E:454:GLU:HB3	1:E:477:TYR:CE1	2.14	0.82
1:C:454:GLU:HB3	1:C:477:TYR:CE1	2.14	0.82
1:D:635:PHE:CE1	1:D:661:LEU:HB2	2.15	0.82
1:A:454:GLU:HB3	1:A:477:TYR:CE1	2.14	0.81
1:F:635:PHE:CE1	1:F:661:LEU:HB2	2.15	0.81
1:B:635:PHE:CE1	1:B:661:LEU:HB2	2.15	0.80
1:C:454:GLU:HB3	1:C:477:TYR:CD1	2.33	0.63
1:A:454:GLU:HB3	1:A:477:TYR:CD1	2.33	0.63
1:E:454:GLU:HB3	1:E:477:TYR:CD1	2.33	0.62
1:D:635:PHE:CZ	1:D:661:LEU:HB2	2.35	0.62
1:F:635:PHE:CZ	1:F:661:LEU:HB2	2.35	0.61
1:B:635:PHE:CZ	1:B:661:LEU:HB2	2.35	0.61
1:A:452:LYS:HB3	1:A:477:TYR:OH	2.05	0.57
1:C:452:LYS:HB3	1:C:477:TYR:OH	2.04	0.57
1:A:98:LYS:NZ	1:F:100:ASP:OD2	2.38	0.57
1:E:452:LYS:HB3	1:E:477:TYR:OH	2.04	0.57
1:E:645:LYS:HD2	1:E:647:TRP:CZ2	2.41	0.56
1:E:98:LYS:NZ	1:D:100:ASP:OD2	2.38	0.56

Continued on next page...

CONFIDENTIAL VALIDATION REPORT

Continued from previous page...

Atom-1	Atom-2	Interatomic distance (Å)	Clash overlap (Å)
1:B:100:ASP:OD2	1:C:98:LYS:NZ	2.38	0.56
1:A:645:LYS:HD2	1:A:647:TRP:CZ2	2.41	0.55
1:C:645:LYS:HD2	1:C:647:TRP:CZ2	2.41	0.55
1:F:345:ARG:CG	1:F:346:ARG:NH1	2.70	0.55
1:B:345:ARG:CG	1:B:346:ARG:NH1	2.70	0.54
1:D:345:ARG:CG	1:D:346:ARG:NH1	2.70	0.54
1:F:341:TRP:CZ2	1:F:345:ARG:HD3	2.43	0.54
1:B:341:TRP:CZ2	1:B:345:ARG:HD3	2.43	0.53
1:D:341:TRP:CZ2	1:D:345:ARG:HD3	2.43	0.52
1:D:345:ARG:HG3	1:D:346:ARG:NH1	2.25	0.52
1:B:345:ARG:HG3	1:B:346:ARG:NH1	2.25	0.51
1:F:345:ARG:HG3	1:F:346:ARG:NH1	2.25	0.51
1:C:728:PHE:CE1	1:C:749:ARG:HG2	2.47	0.50
1:E:24:TRP:HE1	1:E:339:THR:HG1	1.60	0.50
1:E:728:PHE:CE1	1:E:749:ARG:HG2	2.47	0.49
1:A:728:PHE:CE1	1:A:749:ARG:HG2	2.47	0.49
1:E:647:TRP:O	1:E:649:ASN:ND2	2.46	0.49
1:F:635:PHE:CE1	1:F:661:LEU:CB	2.93	0.49
1:C:774:GLY:O	1:C:780:LYS:NZ	2.46	0.48
1:A:647:TRP:O	1:A:649:ASN:ND2	2.46	0.48
1:A:774:GLY:O	1:A:780:LYS:NZ	2.46	0.48
1:E:774:GLY:O	1:E:780:LYS:NZ	2.46	0.48
1:C:647:TRP:O	1:C:649:ASN:ND2	2.46	0.47
1:D:635:PHE:CE1	1:D:661:LEU:CB	2.93	0.47
1:A:622:LYS:HB3	1:A:647:TRP:CD1	2.49	0.47
1:A:647:TRP:HB3	1:A:670:ASN:HB3	1.97	0.47
1:A:433:PHE:CD2	1:A:434:MET:HG2	2.50	0.47
1:C:622:LYS:HB3	1:C:647:TRP:CD1	2.49	0.47
1:A:24:TRP:HE1	1:A:339:THR:HG1	1.63	0.47
1:C:433:PHE:CD2	1:C:434:MET:HG2	2.50	0.47
1:E:433:PHE:CD2	1:E:434:MET:HG2	2.50	0.46
1:E:622:LYS:HB3	1:E:647:TRP:CD1	2.49	0.46
1:C:24:TRP:HE1	1:C:339:THR:HG1	1.63	0.46
1:C:585:ASN:OD1	1:C:585:ASN:N	2.47	0.46
1:E:685:ARG:NH1	1:E:706:LEU:O	2.49	0.46
1:D:502:PHE:HB2	1:D:507:GLU:OE2	2.16	0.46
1:A:585:ASN:N	1:A:585:ASN:OD1	2.47	0.46
1:F:118:LEU:O	1:F:119:HIS:ND1	2.49	0.46
1:C:647:TRP:HB3	1:C:670:ASN:HB3	1.97	0.46
1:E:647:TRP:HB3	1:E:670:ASN:HB3	1.97	0.46
1:F:502:PHE:HB2	1:F:507:GLU:OE2	2.16	0.45

Continued on next page...

CONFIDENTIAL VALIDATION REPORT

Continued from previous page...

Atom-1	Atom-2	Interatomic distance (Å)	Clash overlap (Å)
1:B:345:ARG:HG2	1:B:346:ARG:NH1	2.32	0.45
1:A:682:PHE:HA	1:A:707:LEU:HD21	1.99	0.45
1:E:682:PHE:HA	1:E:707:LEU:HD21	1.99	0.45
1:C:682:PHE:HA	1:C:707:LEU:HD21	1.99	0.45
1:D:118:LEU:O	1:D:119:HIS:ND1	2.49	0.45
1:B:118:LEU:O	1:B:119:HIS:ND1	2.49	0.45
1:F:21:LYS:HA	1:F:22:PRO:HD3	1.89	0.45
1:B:635:PHE:CE1	1:B:661:LEU:CB	2.93	0.44
1:A:685:ARG:NH1	1:A:706:LEU:O	2.49	0.44
1:C:685:ARG:NH1	1:C:706:LEU:O	2.49	0.44
1:E:585:ASN:N	1:E:585:ASN:OD1	2.47	0.44
1:B:502:PHE:HB2	1:B:507:GLU:OE2	2.16	0.44
1:C:728:PHE:CD1	1:C:749:ARG:HG3	2.53	0.44
1:A:728:PHE:CD1	1:A:749:ARG:HG3	2.53	0.44
1:F:345:ARG:HG2	1:F:346:ARG:NH1	2.32	0.44
1:D:345:ARG:HG2	1:D:346:ARG:NH1	2.32	0.43
1:E:129:VAL:HG13	1:E:325:TYR:CE1	2.54	0.43
1:E:728:PHE:CD1	1:E:749:ARG:HG3	2.53	0.43
1:C:129:VAL:HG13	1:C:325:TYR:CE1	2.53	0.43
1:C:433:PHE:H	1:C:433:PHE:HD1	1.67	0.43
1:C:728:PHE:CE1	1:C:749:ARG:CG	3.02	0.43
1:A:433:PHE:HD1	1:A:433:PHE:H	1.67	0.43
1:B:682:PHE:C	1:B:682:PHE:CD1	2.92	0.42
1:E:102:ASP:OD1	1:E:103:ARG:N	2.52	0.42
1:D:682:PHE:C	1:D:682:PHE:CD1	2.92	0.42
1:A:129:VAL:HG13	1:A:325:TYR:CE1	2.54	0.42
1:D:502:PHE:HB2	1:D:507:GLU:CD	2.40	0.42
1:E:728:PHE:CE1	1:E:749:ARG:CG	3.02	0.42
1:F:682:PHE:C	1:F:682:PHE:CD1	2.92	0.42
1:A:728:PHE:CE1	1:A:749:ARG:CG	3.02	0.41
1:D:341:TRP:CH2	1:D:345:ARG:HD3	2.56	0.41
1:A:728:PHE:HB3	1:A:750:VAL:HG23	2.02	0.41
1:C:102:ASP:OD1	1:C:103:ARG:N	2.52	0.41
1:E:728:PHE:HB3	1:E:750:VAL:HG23	2.02	0.41
1:F:502:PHE:HB2	1:F:507:GLU:CD	2.40	0.41
1:E:433:PHE:HD1	1:E:433:PHE:H	1.67	0.41
1:A:102:ASP:OD1	1:A:103:ARG:N	2.52	0.41
1:B:502:PHE:HB2	1:B:507:GLU:CD	2.40	0.41
1:F:119:HIS:HA	1:F:288:ASN:CG	2.41	0.41
1:B:341:TRP:CH2	1:B:345:ARG:HD3	2.56	0.41
1:B:119:HIS:HA	1:B:288:ASN:CG	2.41	0.41

Continued on next page...

CONFIDENTIAL VALIDATION REPORT

Continued from previous page...

Atom-1	Atom-2	Interatomic distance (Å)	Clash overlap (Å)
1:E:730:CYS:SG	1:E:731:ARG:N	2.94	0.41
1:F:341:TRP:CH2	1:F:345:ARG:HD3	2.56	0.41
1:E:593:LEU:HB2	1:E:613:LEU:HD21	2.03	0.41
1:A:730:CYS:SG	1:A:731:ARG:N	2.94	0.40
1:C:728:PHE:HB3	1:C:750:VAL:HG23	2.02	0.40
1:C:730:CYS:SG	1:C:731:ARG:N	2.94	0.40
1:D:119:HIS:HA	1:D:288:ASN:CG	2.42	0.40

There are no symmetry-related clashes.

5.3 Torsion angles (i)

5.3.1 Protein backbone (i)

In the following table, the Percentiles column shows the percent Ramachandran outliers of the chain as a percentile score with respect to all PDB entries followed by that with respect to all EM entries.

The Analysed column shows the number of residues for which the backbone conformation was analysed, and the total number of residues.

Mol	Chain	Analysed	Favoured	Allowed	Outliers	Percentiles	
1	A	700/810 (86%)	641 (92%)	58 (8%)	1 (0%)	53	87
1	B	719/810 (89%)	670 (93%)	47 (6%)	2 (0%)	43	80
1	C	700/810 (86%)	641 (92%)	58 (8%)	1 (0%)	53	87
1	D	719/810 (89%)	670 (93%)	47 (6%)	2 (0%)	43	80
1	E	700/810 (86%)	641 (92%)	58 (8%)	1 (0%)	53	87
1	F	719/810 (89%)	670 (93%)	47 (6%)	2 (0%)	43	80
All	All	4257/4860 (88%)	3933 (92%)	315 (7%)	9 (0%)	53	84

All (9) Ramachandran outliers are listed below:

Mol	Chain	Res	Type
1	B	617	GLN
1	F	617	GLN
1	D	617	GLN
1	A	591	ALA
1	E	591	ALA

Continued on next page...

CONFIDENTIAL VALIDATION REPORT

Continued from previous page...

Mol	Chain	Res	Type
1	C	591	ALA
1	B	167	PRO
1	F	167	PRO
1	D	167	PRO

5.3.2 Protein sidechains (i)

In the following table, the Percentiles column shows the percent sidechain outliers of the chain as a percentile score with respect to all PDB entries followed by that with respect to all EM entries.

The Analysed column shows the number of residues for which the sidechain conformation was analysed, and the total number of residues.

Mol	Chain	Analysed	Rotameric	Outliers	Percentiles	
1	A	631/749 (84%)	630 (100%)	1 (0%)	94	96
1	B	630/749 (84%)	629 (100%)	1 (0%)	94	96
1	C	631/749 (84%)	630 (100%)	1 (0%)	94	96
1	D	630/749 (84%)	629 (100%)	1 (0%)	94	96
1	E	631/749 (84%)	630 (100%)	1 (0%)	94	96
1	F	630/749 (84%)	629 (100%)	1 (0%)	94	96
All	All	3783/4494 (84%)	3777 (100%)	6 (0%)	94	96

All (6) residues with a non-rotameric sidechain are listed below:

Mol	Chain	Res	Type
1	A	647	TRP
1	B	345	ARG
1	E	647	TRP
1	F	345	ARG
1	C	647	TRP
1	D	345	ARG

Some sidechains can be flipped to improve hydrogen bonding and reduce clashes. All (3) such sidechains are listed below:

Mol	Chain	Res	Type
1	A	499	HIS
1	E	499	HIS
1	C	499	HIS

CONFIDENTIAL VALIDATION REPORT

5.3.3 RNA (i)

There are no RNA molecules in this entry.

5.4 Non-standard residues in protein, DNA, RNA chains (i)

There are no non-standard protein/DNA/RNA residues in this entry.

5.5 Carbohydrates (i)

There are no carbohydrates in this entry.

5.6 Ligand geometry (i)

There are no ligands in this entry.

5.7 Other polymers (i)

There are no such residues in this entry.

5.8 Polymer linkage issues (i)

There are no chain breaks in this entry.

CONFIDENTIAL VALIDATION REPORT
