# Supplementary material for: Structure of the human volume regulated anion channel
Source: eLife. 2018 Aug 10;7:e38461. doi: 10.7554/eLife.38461 (PMC6086657; doi:10.7554/eLife.38461)
Supplement: Transparent reporting form [file elife-38461-transrepform.docx]

***eLife’s* transparent reporting form**

We encourage authors to provide detailed information *within their submission* to facilitate the interpretation and replication of experiments. Authors can upload supporting documentation to indicate the use of appropriate reporting guidelines for health-related research (see [EQUATOR Network](http://www.equator-network.org/%20)), life science research (see the [BioSharing Information Resource](https://biosharing.org/)), or the [ARRIVE guidelines](http://www.plosbiology.org/article/info:doi/10.1371/journal.pbio.1000412) for reporting work involving animal research. Where applicable, authors should refer to any relevant reporting standards documents in this form.

If you have any questions, please consult our Journal Policies and/or contact us: [editorial@elifesciences.org](mailto:editorial@elifesciences.org).

**Sample-size estimation**

- You should state whether an appropriate sample size was computed when the study was being designed
- You should state the statistical method of sample size computation and any required assumptions
- If no explicit power analysis was used, you should describe how you decided what sample (replicate) size (number) to use

Please outline where this information can be found within the submission (e.g., sections or figure legends), or explain why this information doesn’t apply to your submission:

For electrophysiological experiments, sample sizes represent the number of cells tested in whole cell recordings. The number of cells tested was determined based on the magnitude of the effect observed and the variance among data points. The sizes were chosen based on consistency of data across conditions and multiple experiments.

**Replicates**

- You should report how often each experiment was performed
- You should include a definition of biological versus technical replication
- The data obtained should be provided and sufficient information should be provided to indicate the number of independent biological and/or technical replicates
- If you encountered any outliers, you should describe how these were handled
- Criteria for exclusion/inclusion of data should be clearly stated
- High-throughput sequence data should be uploaded before submission, with a private link for reviewers provided (these are available from both GEO and ArrayExpress)

Please outline where this information can be found within the submission (e.g., sections or figure legends), or explain why this information doesn’t apply to your submission:

Electrophysiological experiments were reproduced according to the sample size as indicated in each figure. The recordings and parameters were reproducible across multiple days and transfection batches. Each experiment was performed on cells from at least 3 separate transfections (for WT in Cl^-^ solutions, 6 separate experiments; WT in Iodide solutions, 4 separate experiments; T5C in 5 separate experiments using Cl^-^ and I^-^; T5R in 3 separate experiments in Cl^-^ and 3 separate experiments in I^-^; R103F in 3 separate experiments. Three separate transfections were performed to acquire LRRC8A only vs vector data.

Definition of biological versus technical replication. Electrophysiological experiments to characterize mutant vs WT channels (e.g., extracellular ATP block, MTSES block, V_rev_) were routinely done by interleaving the cells to be compared to avoid unavoidable differences imposed on the protocol during the course of the experiment such as the time cells were removed from the incubator and fluctuations in room temperature. An agar bridge was used for all experiments and electrode wires were chloride coated routinely (2-3 times per week) during the course of these experiments to avoid drift.

Information is provided throughout the manuscript in figure legends indicating the number of independent biological and/or technical replicates.

Outliers. One cell expressing WT + LRRC8C was not included in the data set because the capacitance suggested it was not a single cell and the V_rev_ of the hypotonic solution induced current was 16.5 mV which was >2 standard deviations beyond the mean (which included this data point).

Criteria for exclusion/inclusion of data. No data were excluded unless contaminating leak currents obscured the recordings. When the HeLa LRRC8^-/-^ cells revealed a noisy, strongly inwardly rectifying current (its biophysical and pharmacological characteristics differed from VRAC), data acquisition was aborted.

**Statistical reporting**

- Statistical analysis methods should be described and justified
- Raw data should be presented in figures whenever informative to do so (typically when N per group is less than 10)
- For each experiment, you should identify the statistical tests used, exact values of N, definitions of center, methods of multiple test correction, and dispersion and precision measures (e.g., mean, median, SD, SEM, confidence intervals; and, for the major substantive results, a measure of effect size (e.g., Pearson's r, Cohen's d)
- Report exact p-values wherever possible alongside the summary statistics and 95% confidence intervals. These should be reported for all key questions and not only when the p-value is less than 0.05.

Please outline where this information can be found within the submission (e.g., sections or figure legends), or explain why this information doesn’t apply to your submission:

For quality assessment of EM structures, Fourier shell correlation between independently refined half datasets is shown in Figure 1-figure supplement 3d. Additionally, we report separate EMRinger scores and map-model cross correlation for the entire model (Swell1), the pore-forming domains, and the LRR domains.

For electrophysiology experiments, the exact sample size (N) for each experimental group/condition is given as a discrete number and unit of measurement (cells tested from the number of separate cultures/transfections). The number of times each experiment was replicated is included in the figure legend (also see above). Measurements were taken from a single cell per coverslip, therefore no cell was pre-exposed to hypotonic solution. Since the datapoints fall within a Gaussian distribution, the Student’s *t*-test was used to compare two datasets. Means ± standard error of the mean (s.e.m.) for N samples is shown with clear error bars and N shown associated with the error bar. P values are given as exact values whenever possible and with confidence intervals noted.

(For large datasets, or papers with a very large number of statistical tests, you may upload a single table file with tests, Ns, etc., with reference to sections in the manuscript.)

**Group allocation**

- Indicate how samples were allocated into experimental groups (in the case of clinical studies, please specify allocation to treatment method); if randomization was used, please also state if restricted randomization was applied
- Indicate if masking was used during group allocation, data collection and/or data analysis

Please outline where this information can be found within the submission (e.g., sections or figure legends), or explain why this information doesn’t apply to your submission:

For patch clamp recordings, samples were grouped based on the cDNAs of interest transfected into the cells. The investigators were not blinded to group allocation.

**Additional data files (“source data”)**

- We encourage you to upload relevant additional data files, such as numerical data that are represented as a graph in a figure, or as a summary table
- Where provided, these should be in the most useful format, and they can be uploaded as “Source data” files linked to a main figure or table
- Include model definition files including the full list of parameters used
- Include code used for data analysis (e.g., R, MatLab)
- Avoid stating that data files are “available upon request”

Please indicate the figures or tables for which source data files have been provided:

The cryo-EM map of human SWELL1 was deposited into the Electron Microscopy Data Bank with accession code 7935. The atomic model of human SWELL1 was deposited into the Protein Data Bank with PDB ID 6DJB.
